# Assessing the quality of data for selected reproductive health indicators in designated public health facilities in Bangladesh

**DOI:** 10.7189/jogh.14.04259

**Published:** 2024-12-06

**Authors:** Fauzia Akhter Huda, Meftah Uddin Mahmud, Tanjeena Tahrin Islam, Salma Akter, Sadia Fatema Kabir, Md Shahadat Hossain, Shah Ali Akbar Ashrafi, M Naser Uddin, Farhana Habib, Sharon Kim Gibbons, Onikepe O Owolabi

**Affiliations:** 1 Maternal and Child Health Division, icddr,b, Dhaka, Bangladesh; 2 Health System and Population Studies Division, icddr,b, Dhaka, Bangladesh; 3 Department of Data Science, RMIT University, Melbourne, Victoria, Australia; 4 South Asia Field Epidemiology and Technology Network, Dhaka, Bangladesh; 5 MIS Unit, Directorate General of Health Service, Dhaka, Bangladesh; 6 IEM, Directorate General of Family Planning, Dhaka, Bangladesh; 7 Data Impact Program Bangladesh, Vital Strategies, Dhaka, Bangladesh; 8 Vital Strategies, New York, USA; 9 Guttmacher Institute, New York, USA

## Abstract

**Background:**

An effective health management information system plays a pivotal role in evidence-based decision-making and strengthening health service delivery in a country. The Directorate General of Health Services and the Directorate General of Family Planning of Bangladesh have adopted digital health management information system platforms named district health information system and management information system, respectively. Despite its significance, health management information system data has numerous issues, such as missing values, inaccuracies, lack of internal consistency, and the presence of outliers. This study aims to assess the data quality of reproductive health indicators in the health management information system of the Directorate General of Health Services and the Directorate General of Family Planning.

**Methods:**

The study examined two aspects of data quality: a) completeness of data, subdivided into completeness of facility reporting (report submission rate) and completeness of indicator data (presence of missing values); b) internal consistency of reported data, subdivided into presence of outliers, inter-indicator consistency, and consistency between reported data and original records (accuracy rate). The study utilised retrospective monthly data gathered from July 2021 to June 2022, covering 21 reproductive health indicators. Multi-stage cluster sampling was employed to select 112 health facilities for data collection, including 48 facilities from Directorate General of Health Services and 64 from Directorate General of Family Planning, representing various administrative levels across the country.

**Result:**

The report submission rate for Directorate General of Health Services facilities was 98%, while for the Directorate General of Family Planning facilities, it was 86%. However, 35% of data points were missing in the district health information system server of Directorate General of Health Services, whereas no missing values were observed in the management information system server of Directorate General of Family Planning. Less than 3% of outliers were detected in the server data of both directorates. Inter-indicator consistency was maintained at a high rate of 98% in health facilities under both directorates. The accuracy of reported data varied across indicators and facility types: Directorate General of Health Services facilities showed accuracy rates ranging between 75 and 92%, with an aggregated rate of 86%. Different tiers of the Directorate General of Family Planning facilities had accuracy rates ranging from 92 to 96%.

**Conclusion:**

This research emphasises the significance of rectifying missing values, ensuring consistency, and improving reporting systems, with a particular focus on lower-tier health facilities, to enhance the validity and reliability of reproductive health data in Bangladesh.

The World Health Organization (WHO) emphasises that an effective Health Management Information System (HMIS) ensures the generation, analysis, dissemination, and utilisation of accurate and timely data on health determinants, health system performance, and health status [1]. Globally, health management information system plays a crucial role in evidence-based decision-making and improving health service delivery [2,3], being one of WHO’s six core components for Health Systems Strengthening (HSS) [1]. Improving health management information system enhances health information reliability, essential for public health reform strategies in developing nations [4]. Many low- and middle-income countries (LMICs) implement national health management information system for routine facility-based health care data collection [5,6], aligning with Sustainable Development Goals (SDGs) and other global initiatives for monitoring progress and enabling adjustments [7–9].

Over the past 50 years, Bangladesh has significantly advanced health and family planning (FP) services, notably improving several health indicators like infant mortality rate (IMR), maternal mortality ratio (MMR), and under-5 mortality rate (U5MR) [10–14]. Initiatives such as emergency obstetric care (EmOC) and maternal health voucher schemes have been introduced to improve women’s reproductive health [13,15,16]. Despite progress, significant portions of the population lack access to adequate health care [12,17–20], with around half of childbirths still occurring at home, posing risks to the mother and new-born’s health [10,18,20].

Quality health management information system data on maternal and newborn health care are essential for identifying gaps in current facility-based management, developing effective interventions, and tracking progress [21]. Recognising the growing importance of health management information system, many countries have implemented the District Health Information System (DHIS2), an open-source web-based software platform [22]. This software facilitates the continuous gathering, consolidation, and visualisation of health data across all levels of the system [23].

Studies from Uganda and Kenya demonstrates that district health information system deployment has improved reporting on immunisation coverage, antenatal care (ANC) visits, and facility delivery rates [14,24,25]. Evidence from Laos shows that effective application of district health information system on maternal, new-born and child health (MNCH) surveillance data has enhanced service delivery [26]. Since its implementation, district health information system has been adopted in more than 76 countries worldwide for gathering and analysing health data [27].

In Bangladesh, the Ministry of Health and Family Welfare (MoH&FW) oversees health, family planning, and nutrition programmes [28] through two directorates: Directorate General of Health Services (DGHS) and Directorate General of Family Planning (DGFP) [29]. Directorate General of Health Services utilises district health information system as its health management information system, while Directorate General of Family Planning has its own online-based Management Information System (MIS) [13].

Despite its significance, data from health management information system face numerous challenges, including missing values, inaccuracies, biases, unidentified sources, and underutilisation of tools [23,30–39]. Moreover, health care workers encounter difficulties such as inadequate skills, heavy workload, lack of effective monitoring systems, and the absence of standard guidelines regarding health management information system tools and indicators [31]. These challenges may compromise the effectiveness of achieving health goals at both the national and sub-national levels [1]. Therefore, conducting regular Data Quality Assurance (DQA) is imperative to identify potential gaps and opportunities for improvement in meeting defined data standards, such as completeness, accuracy, consistency, and timeliness [32,40,41].

This study aims to assess the data quality of reproductive health-related indicators in Bangladesh’s health management information system. It focuses on core maternal and child health and family planning (MCH-FP) indicators, within the context of sexual and reproductive health and rights (SRHR), aligning with both the SDGs and Bangladesh's internal strategies for women’s health and gender equity. This approach ensures nuanced policy interventions for improving maternal health outcomes while addressing broader reproductive health issues.

## METHODS

### Study design

This study followed World Health Organization’s (WHO) data quality assurance guidelines and utilised secondary data sources [32,40,41]. The assessment involved a desk review, selection of indicators and health facilities, data collection and analysis, and a stakeholder consultation workshop. Although it utilised secondary data, the study effectively functions as a primary investigation of these sources.

### Data collection

After reviewing literature and assessing health facility services, initially, six indicators focusing on women’s health, specifically menstrual regulation (MR), its complications, and post-abortion care (PAC), were selected to address study objectives. Meetings with key stakeholders from Directorate General of Health Services and Directorate General of Family Planning led to the addition of 15 more indicators, covering modern contraceptive use, childbirth details (including delivery mode, outcomes, and complications), postpartum family planning, and maternal mortality. This resulted to a final set of 21 indicators for the DQA.

Monthly data segregated from July 2021 to June 2022 (12-month period), covering the selected 21 indicators, was collected from the facility registers of the chosen health facilities and the district health information system and management information system platforms, where the facilities upload their monthly reports. To adhere to WHO toolkits and study objectives, paper-based data collection tools were developed for extracting information from health facility registers, following field testing to ensure effectiveness and accuracy.

Bangladesh is divided into eight administrative divisions, each further subdivided into districts, then into upazilas (sub-districts), unions, and villages. At the tertiary health care level, each division hosts a Medical College Hospital (MCH) under Directorate General of Health Services. At the secondary level, each district has a District Hospital (DH) under Directorate General of Health Services and a Maternal and Child Welfare Center (MCWC) under Directorate General of Family Planning.

Moving down to the primary level, each upazila operates an Upazila Health Complex (UHC) under Directorate General of Health Services, serving as the initial point of referral in the country’s health care system. Additionally, each upazila houses a Sadar Clinic and/or a Maternal and Child Health-Family Planning (MCH-FP) unit under Directorate General of Family Planning. Union Health & Family Welfare Centers (UH&FWC) under Directorate General of Family Planning and Union Sub-Centers under Directorate General of Health Services provide services at the union level.

**Figure 1:**
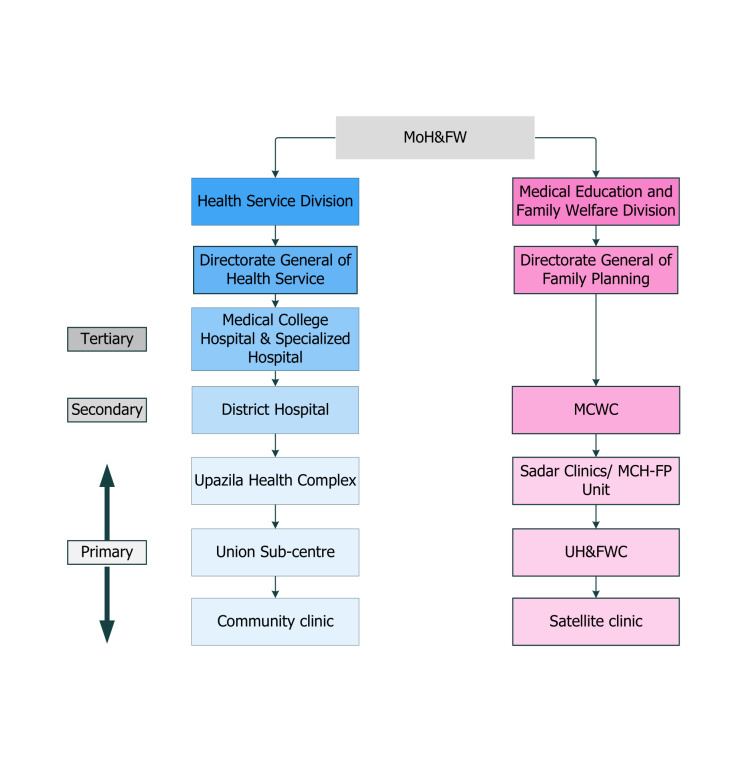
Organogram of health service delivery system in Bangladesh. MoH&FW – Ministry of Health and Family Welfare, MCWC – Maternal and Child Welfare Center, MCH-FP – Maternal and Child Health-Family Planning, UH&FWC – Union Health & Family Welfare Center

At the grassroots level in rural Bangladesh, Community Clinics (CC) under Directorate General of Health Services and satellite clinics under Directorate General of Family Planning cater to the health care needs of different villages. The distribution of these health facilities across administrative units are illustrated in **Figure 1**.

Primary data collection in Bangladesh’s health facilities is conducted using registers and forms. At the end of each month, data from these facilities are compiled and summarised in monthly reports. Subsequently, these reports are entered into either district health information system or management information system, depending on the respective directorate.

Employing a multi-stage cluster sampling approach, health facilities were selected for Data Quality Assurance (DQA) from all eight divisions across the country. The inclusion criteria for this study encompassed public health facilities at various tiers operated by either the Directorate General of Health Services or the Directorate General of Family Planning that provide reproductive health services. Conversely, private health facilities and non-functional public health facilities were excluded from the study. The list of public health facilities at different administrative levels was obtained from the government health facility registry, which served as the sampling frame for the study [42].

Through simple random sampling, we selected two districts from each division to represent district-level health facilities. Out of the 16 selected districts, two had medical college hospitals and 14 had district hospitals, with one of each type per district. Additionally, each of these 16 districts had one maternal and child welfare centres. Therefore, a total of 32 facilities were selected at the district level.

Subsequently, we randomly identified 16 upazilas (sub-districts) from the selected districts, one from each district. Each selected upazila had one upazila health complex, one Sadar clinic, and one MCH-FP unit, totalling 48 facilities included in the sample.

Thereafter, we randomly picked 16 union health & family welfare centres from among these upazilas to assess data quality at the union level health facilities. Finally, from each of the unions selected in the previous stage, one community clinic was randomly chosen.

Therefore, a total of 112 health facilities were selected for the data quality assurance, comprising 48 facilities under Directorate General of Health Services (including two medical college hospitals, 14 district hospitals, 16 upazila health complexes, and 16 community clinics), and 64 facilities under Directorate General of Family Planning (including 16 maternal and child welfare centres, 16 sadar clinics, 16 MCH-FP units, and 16 union health & family welfare centres). The lists of selected facilities under Directorate General of Health Services and Directorate General of Family Planning are provided in Tables S1–2 in the **Online Supplementary Document**.

It is essential to note that health facilities at different tiers provide varying levels of services, leading to differences in indicators across primary, secondary, and tertiary levels. This underscores the need to tailor indicator selection to the specific context and services provided by each level of health facility.

Two teams, each consisting of two researchers, visited the selected health facilities to collect data from facility registers. In addition to quantitative data collection, the researchers conducted informal, unstructured discussions with key staffs during these visits. Verbal consent was obtained, and although these discussions were not recorded electronically, detailed notes were taken to capture the key points while maintaining anonymity.

Information presented in **Table 1** outlines the list of indicators, along with the levels and number of facilities included in the DQA analysis, providing specific or relevant services.

**Table 1:** List of indicators, and number of the health facilities provide relevant services and expected to report data for the indicators

Indicators/ data elements	Number of facilities							
	**Tertiary level**	**Secondary level**		**Primary level**				
	**Division/district level**	**District level**		**Sub-district level**			**Union level**	**Village level**
	Medical college hospital*	District Hospital*	**MCWC†**	UHC*	**Sadar Clinic†**	**MCH-FP†**	**UH&FWC†**	Community Clinic*
Pill	–	–	15	–	12	5	14	9
Condom	–	–	14	–	12	5	14	8
Injectable	–	–	14	–	12	4	14	6
IUD	–	–	15	–	12	4	13	–
Implant	–	–	15	–	12	4	9	–
Tubectomy/ligation (female)	–	–	15	–	12	4	7	–
Vasectomy (male)	–	–	14	–	12	4	7	–
Manual vacuum aspiration	–	–	14	–	11	3	5	–
Menstrual regulation with medication	–	–	14	–	10	1	5	–
Post-abortion care	–	–	14	–	10	4	5	–
Normal vaginal delivery	2	13	15	15	10	2	12	2
Caesarean section	2	13	14	10	8	1	0	–
Forceps/vacuum/breech delivery	2	13	–	15	–	–	–	–
Livebirth	2	13	15	15	10	2	12	2
Stillbirth	2	13	15	15	10	2	12	2
Pre-eclampsia/eclampsia	2	13	15	15	10	2	12	–
Postpartum haemorrhage	2	13	15	15	10	2	12	–
Prolonged/obstructed labour	2	13	–	15	–	–	–	–
Septic abortion	2	13	–	15	–	–	–	–
Postpartum family planning	2	13	–	15	–	–	–	–
Maternal death	2	13	15	15	10	2	12	2

MCWC – Maternal and Child Welfare Center, UHC – Upazila Health Complex, MCH-FP – Maternal and Child Health-Family Planning, UH&FWC – Union Health & Family Welfare Center

*Directorate General of Health Services operated health facilities.

†Directorate General of Family Planning operated health facilities.

### Data analysis

Considering WHO guidelines, the study assessed two aspects of the data quality: completeness of data and internal consistency of reported data [32]. The data were analysed using STATA Version 15.0 (StataCorp LLC, College Station, Texas, USA). To ensure valid comparisons and account for differences in service types across various administrative levels, we segregated the databased on health facility types.

### Completeness of data

Data completeness has two different aspects: facility reporting (report submission rate), and completeness of indicators. Facility reporting is calculated by dividing the number of monthly reports received by the server by the expected yearly reports [32]. WHO recommends a 75% reporting rate to be acceptable [32].

Indicator completeness referred to the percentage of non-missing indicator values, focusing on the completeness of specific data elements [32]. Initially computed per facility, it’s the count of non-missing values divided by the expected number of values. Summary statistics are then aggregated. Indicator completeness was measured for those months when monthly reports were submitted in the database. WHO suggests accepting a maximum of 25% missing values in the reported data [32].

### Internal consistency of reported data

The analysis of internal consistency in reported data involved three main approaches. First, outliers, defined as significantly distinct indicator values compared to other monthly data per facility, were identified. It’s important to note that outliers may not necessarily indicate errors but require further exploration and verification during data quality checks. Moderate outliers were defined as monthly values that deviated from the facility’s monthly mean value of the indicator by at least two standard deviations, while extreme outliers deviated by at least three standard deviations.

Second, inter-indicator consistency (IIC) was examined by comparing the sum of normal vaginal deliveries (NVD), caesarean sections (C/S), and forceps/vacuum/breech deliveries with the total live births and stillbirths. As per WHO guidelines, differences of less than 10% between these values were accepted as indicative of the presence of inter-indicator consistency [32]. Summary statistics were then derived from facility-wide findings.

Finally, the consistency of reported data with original records, termed the accuracy rate, was assessed. This approach aimed to identify accurately reported, under-reported, and over-reported values in the server compared to the source data (facility register data). This analysis was conducted separately for each indicator and each month. This involved comparing reported values with data extracted from facility registers. Values within ±10% of register data were deemed accurate. Deviations greater or lesser than 10% indicated over-reporting or under-reporting, respectively [41]. WHO recommends at least 75% accuracy in the server database [41].

The qualitative data from the informal, unstructured discussions were initially recorded anonymously in hardcopy by the research team members. These notes were then transcribed into Bangla, the local language, and subsequently translated into English. Thematic analysis was performed to understand the reporting process and identify challenges associated with daily reporting activities.

A stakeholder consultation workshop was organised with key relevant stakeholders from Directorate General of Health Services and Directorate General of Family Planning. Preliminary findings from the data quality assurance were shared with the key stakeholders for validation and to draw recommendations. Gaps identified in the data quality assurance and the way forward have been discussed with the stakeholders through a consultative process.

## RESULTS

Of the 48 Directorate General of Health Services facilities initially selected, four community clinics were found to be non-functional and permanently closed during the field visit. Reports from these facilities were not available on the district health information system server, so they were excluded from the analysis according to the guidelines [32,40,41]. In addition, two community clinics, one upazila health complex, and one district hospital lacked paper-based reporting systems, which rendered data collection infeasible. Therefore, these eight facilities were excluded, leaving 40 Directorate General of Health Services facilities for analysis.

Similarly, from the initial 64 Directorate General of Family Planning facilities, one MCH-FP unit and one union health & family welfare centre were found to be non-functional and permanently closed, and seven MCH-FP units were merged with Sadar Clinics. This led to excluding nine facilities, resulting in 55 Directorate General of Family Planning facilities available for analysis.

### Completeness of data

#### Completeness of facility reporting

In all tiers of Directorate General of Health Services facilities, except for community clinics, the report submission rate stood at a commendable 100%. Over the 12-month reporting period, the completeness of facility reporting for community clinics reached 90%, with 108 reports submitted out of the expected 120. Overall, across all tiers of Directorate General of Health Services facilities, 98% (468 out of the expected 480) of monthly reports were successfully submitted to district health information system, surpassing the WHO cut off value.

Among the four types of Directorate General of Family Planning facilities, maternal and child welfare centres achieved the highest report submission rate at 94%, closely followed by union health & family welfare centres at 93%. Both Sadar clinics and MCH-FP units under Directorate General of Family Planning exhibited a 75% report submission rate (**Table 2**).

**Table 2:** Completeness of facility reporting by types of facilities*

Facility type	No. of facilities	Expected number of reports	Report submitted	Report not submitted	% of report submitted
DGHS	40	480	468	12	97.5
MCH	2	24	24	0	100.0
District Hospital	13	156	156	0	100.0
UHC	15	180	180	0	100.0
CC	10	120	108	12	90.0
DGFP	55	660	564	96	85.5
MCWC	16	192	180	12	93.8
Sadar Clinic	16	192	144	48	75.0
MCH-FP Unit	8	96	72	24	75.0
UH&FWC	15	180	168	12	93.3

CC – community clinic, DGFP – Directorate General of Family Planning operated health facilities, DGHS – Directorate General of Health Services, MCH **–** Medical College Hospital, MCH-FP – Maternal and Child Health-Family Planning, MCWC – Maternal and Child Welfare Center, UHC – Upazila Health Complex, UH&FWC – Union Health & Family Welfare Center

*World Health Organization recommended cut off value is 75%.

#### Completeness of indicator data

The completeness rates of indicator data across Directorate General of Health Services facilities varied, with an average of 65%, which is 10% below the WHO recommendation. Medical college hospitals had the highest completeness rate at 91%, followed by district hospitals at 77%. Conversely, upazila health complexes and community clinics had lower rates, with upazila health complexes at 50% and community clinics at 67% (**Table 3**). Geographically, it was observed that the completeness rates of indicator data among the Directorate General of Health Services facilities varied between 58 and 74% across the eight divisions (**Table 4**).

**Table 3:** Completeness of indicator data by types of facilities*

Facility type	Expected total records	No. of records submitted with values	Completeness rate (%)	Missing values (%)	No. of facilities	No. of facilities with completeness rate less than cut-off value
DGHS	4270	2764	64.7*	35.3	39	24
MCH	264	241	91.3	8.7	2	0
District Hospital	1716	1320	76.9	23.1	13	5
UHC	1918	953	49.7*	50.3	15	15
CC	372	250	67.2*	32.8	9	4
DGFP	10 848	10 848	100.0	0.0	47	0
MCWC	4032	4032	100.0	0.0	15	0
Sadar Clinic	3060	3060	100.0	0.0	12	0
MCH-FP Unit	924	924	100.0	0.0	6	0
UH&FWC	2832	2832	100.0	0.0	14	0

CC – community clinic, DGFP – Directorate General of Family Planning operated health facilities, DGHS – Directorate General of Health Services, MCH – Medical College Hospital, MCH-FP – Maternal and Child Health-Family Planning, MCWC – Maternal and Child Welfare Center, UHC – Upazila Health Complex, UH&FWC – Union Health & Family Welfare Center

*Completeness is lower than the World Health Organization’ accepted standards. World Health Organization recommended cut off value is 75%.

**Table 4:** Completeness of indicator data by division (Directorate General of Health Services operated facilities)*

Division	Expected Total records	No. of records submitted with values	No. of records with missing values	Completeness rate (%)	Missing values (%)
Barishal	384	285	99	74	26
Chittagong	588	365	223	62	38
Dhaka	576	382	194	66	34
Khulna	636	367	269	58	42
Mymensingh	420	254	166	60	40
Rajshahi	552	354	198	64	36
Rangpur	528	354	174	67	33
Sylhet	588	403	185	69	31
Total	4272	2764	1508	65	35

*World Health Organization recommended cut off value is 75%.

Directorate General of Family Planning facilities achieved 100% completeness, with missing values replaced uniformly with ‘0’ (zero) across all tiers (**Table 3**).

### Internal consistency of reported data

#### Presence of moderate and extreme outliers

Analysis of the current study revealed that both Directorate General of Health Services and Directorate General of Family Planning databases had outliers within the WHO defined acceptable limit. Out of 13 612 reported values (2764 from DGHS and 10 848 from DGFP) across 86 facilities, outlier rates were 2.5% for Directorate General of Health Services and 2.1% for Directorate General of Family Planning. Specifically, 276 (2%) moderate outliers were observed across 75 facilities, and 19 (0.1%) extreme outliers were detected across 15 facilities (**Table 5**). Notably, certain facilities exhibited both moderate and extreme outliers.

**Table 5:** Presence of outliers in the monthly report by types of facilities*

Facility Type	Total records (missing values excluded)	No. of moderate outliers (% of values with moderate outliers)	No. of extreme outliers (% of values with extreme outliers)	Total No. of outliers (% of values with outliers)	No. of facilities	No. of facilities with moderate outliers	No. of facilities with extreme outliers
DGHS	2764	63 (2.3)	5 (0.2)	68 (2.5)	39	30	4
MCH	241	8 (3.3)	0 (0)	8 (3.3)	2	2	0
District Hospital	1320	28 (2.1)	2 (0.2)	30 (2.3)	13	11	2
UHC	953	20 (2.1)	0 (0)	20 (2.1)	15	13	0
CC	250	7 (2.8)	3 (1.2)	10 (4.0)	9	4	2
DGFP	10 848	213 (2)	14 (0.1)	227 (2.1)	47	45	11
MCWC	4032	82 (2.0)	10 (0.2)	92 (2.3)	15	15	7
Sadar Clinic	3060	63 (2.1)	1 (0.03)	64 (2.1)	12	11	1
MCH-FP Unit	924	21 (2.3)	1 (0.1)	22 (2.4)	6	5	1
UH&FWC	2832	47 (1.7)	2 (0.1)	49 (1.7)	14	14	2

CC – community clinic, DGFP – Directorate General of Family Planning operated health facilities, DGHS – Directorate General of Health Services, MCH – Medical College Hospital, MCH-FP – Maternal and Child Health-Family Planning, MCWC – Maternal and Child Welfare Center, UHC – Upazila Health Complex, UH&FWC – Union Health & Family Welfare Center

*World Health Organization recommendation: moderate outlier: ±2–3 standard deviation from mean. Extreme outlier: at least ±3 standard deviation from mean.

#### Inter-indicator consistency (IIC)

The inter-indicator consistency was calculated based on the indicators outlined in the methodology section for facilities of all tiers except community clinics. In community clinics, no indicators were found to exhibit a predictable relationship according to our equation (NVD+C/S+Forceps/vacuum/breech deliveries = Live births + still births). Notably, inter-indicator consistency rates of 98% or higher were observed across all types of Directorate General of Health Services and Directorate General of Family Planning facilities (**Table 6**).

**Table 6:** Inter indicator consistency by types of facilities*

Facility Type	Total records	No. of records with consistency	Inter-indicator consistency rate (%)	No. of facilities	No. of facilities without consistency
DGHS	360	355	98.6	30	5
MCH	24	24	100.0	2	0
District Hospital	156	153	98.1	13	3
UHC	180	178	98.9	15	2
CC	—	—	—	—	—
DGFP	564	560	99.3	47	4
MCWC	180	178	98.9	15	2
Sadar Clinic	144	143	99.3	12	1
MCH-FP Unit	72	72	100.0	6	0
UH&FWC	168	167	99.4	14	1

CC – community clinic, DGFP – Directorate General of Family Planning operated health facilities, DGHS – Directorate General of Health Services, MCH – Medical College Hospital, MCH-FP – Maternal and Child Health-Family Planning, MCWC – Maternal and Child Welfare Center, UHC – Upazila Health Complex, UH&FWC – Union Health & Family Welfare Center

*Inter indicator consistency rate: % of facilities where the relationship between predictable indicators is <10%.

#### Consistency of reported data and original records

Consistency between reported data and the original records was verified for the data available in the databases. Out of 13 612 reported values, the overall data accuracy rate was 92%, meeting the WHO cut-off value requirement of at least 75% accurate values in the facility reports. Specifically, the accuracy rate was 86% for Directorate General of Health Services facilities and 94% for Directorate General of Family Planning facilities (**Table 7**).

**Table 7:** Accuracy, under-reporting, and over-reporting by types of facilities*

Facility Type	Total records (missing values excluded)	Accurate records (n)	Accuracy rate (%)	Under-reported records (n)	Under-reported records (%)	Over-reported records (n)	Over-reported records (%)	Facilities (n)	Facilities with underreported records (n)	Facilities with overreported records (n)
DGHS	2764	2371	85.8	88	3.2	305	11.0	39	16	29
MCH	241	181	75.1	28	11.6	32	13.3	2	2	2
District Hospital	1320	1151	87.2	26	2.0	143	10.8	13	5	10
UHC	953	810	85.0	23	2.4	120	12.6	15	6	12
CC	250	229	91.6	11	4.4	10	4.0	9	3	5
DGFP	10 848	10 148	93.5	267	2.5	433	4.0	47	45	43
MCWC	4032	3760	93.3	115	2.9	157	3.9	15	15	14
Sadar Clinic	3060	2816	92.0	80	2.6	164	5.4	12	11	12
MCH-FP Unit	924	883	95.6	22	2.4	19	2.1	6	6	4
UH&FWC	2832	2689	95.0	50	1.8	93	3.3	14	13	13

CC – community clinic, DGFP – Directorate General of Family Planning operated health facilities, DGHS – Directorate General of Health Services, MCH – Medical College Hospital, MCH-FP – Maternal and Child Health-Family Planning, MCWC – Maternal and Child Welfare Center, UHC – Upazila Health Complex, UH&FWC – Union Health & Family Welfare Center

*Accuracy: difference between source data and server data are <10% of the source data. Under-reporting: server data are ≤10% of source data. Over-reporting: server data are ≥10% of source data.

Among the Directorate General of Health Services facilities, under-reporting and over-reporting of data were identified at rates of 3 and 11%, respectively. Conversely, Directorate General of Family Planning facilities exhibited under-reporting and over-reporting rates of 2 and 4%, respectively.

### Informal discussion with the reporting focal persons

During visits to a total of 112 health facilities, informal discussions were held with only 35 focal persons, comprising 18 from Directorate General of Health Services facilities and 17 from Directorate General of Family Planning facilities. This limited participation was attributed to our visits coinciding with the busy working hours of the focal persons, who were engaged in their regular office tasks. From these discussions, several noteworthy findings emerged.

In Directorate General of Health Services facilities, dedicated focal persons for reporting were typically appointed at sub-district and higher-level facilities, except for community clinics where this responsibility was assumed by service providers. In contrast, Directorate General of Family Planning facilities lacked dedicated reporting focal persons, with Family Welfare Visitors (FWVs) being responsible for monthly reporting at all levels.

A significant number of reporting focal persons, family welfare visitors, and service providers from both the Directorate General of Health Services and Directorate General of Family Planning facilities had not received specific training on data entry or the reporting process in the health management information system. Many community clinics and union-level facilities faced challenges due to a lack of essential resources such as laptops and internet connections, resulting in occasional failures in data submission due to unidentified technical issues with the server. The simultaneous burden of manual data collection and entry into the server posed challenges, especially in facilities with limited human resources.

There was inconsistency in the regular supply of reporting forms, with no budget allocated at the local level for printing forms. Furthermore, a significant absence of documents outlining the standard operational definitions of indicators among service providers or reporting focal persons at both Directorate General of Health Services and Directorate General of Family Planning facilities was highlighted, suggesting a potential gap in understanding and adherence to standardised reporting protocols.

### Stakeholder consultation workshop

A stakeholder consultation workshop was held involving government stakeholders from Directorate General of Health Services and Directorate General of Family Planning, as well as representatives from Vital Strategies. The workshop aimed to share preliminary findings of a study and discuss strategies to address identified bottlenecks in data quality assurance.

Directorate General of Health Services officials acknowledged data quality concerns due to a shortage of reporting focal persons in some health facilities. Although the management information system unit of the Directorate General of Health Services conducts frequent trainings for these focal persons, staff turnover often creates vacancies. Despite improvements since the initial deployment of district health information system (DHIS2), discrepancies persist in the data. Directorate General of Health Services is actively addressing these issues to improve the overall data reporting system and data quality.

Similarly, Directorate General of Family Planning officials noted a shortage of human resources for reporting, particularly at divisional and district levels. The government has approved the recruitment of new staff to address this need. Service providers at union level health facilities face challenges in both service provision and management information system reporting, yet no reporting focal person position has been established for these facilities. Directorate General of Family Planning is transitioning to a paperless reporting system and aims to implement electronic management information system (e-MIS) in all health facilities nationwide by June 2024.

## DISCUSSION

The findings of the current study regarding the completeness rates of reproductive, maternal, new-born, and child health (RMNCH) data from health facilities under both Directorate General of Health Services and Directorate General of Family Planning are crucial for national policy formulation and decision-making, particularly in the context of improving women's health. While meeting the WHO’s standards for overall completeness rates is a positive indicator, the absence of monthly reports from nine facilities, particularly from Directorate General of Family Planning facilities, highlights a significant gap in data submission to the authorities. This lack of reporting directly affects the reliability and accuracy of reproductive, maternal, new-born, and child health data, which are essential for assessing the effectiveness of health programmes and interventions aimed at improving women's health outcomes.

Furthermore, the high presence of missing values among Directorate General of Health Services facilities, especially at sub-district and lower-level facilities, indicates discrepancies in data reporting and underscores the urgent need for improvements in data collection and management practices within the context of women’s health. Similar issues with missing values in district health information system (DHIS2) for maternal and reproductive health indicators have been observed in other middle-income countries in Africa, indicating a broader issue that requires attention [30].

Moreover, lower completeness rates for specific indicators such as forceps/vacuum/breech deliveries, maternal deaths, and septic abortion highlight areas where data quality requires enhancement to accurately reflect the state of maternal and reproductive health. Additionally, except for normal vaginal deliveries and live births, all indicators fell below the WHO’s cut-off point in upazila health complexes, indicating the need for a more in-depth exploration of these indicators within the reporting system of the respective health facilities, particularly concerning women's health. Addressing these challenges and improving data reporting practices are critical steps in advancing women's health outcomes and achieving broader health goals.

In contrast, the absence of missing values in reports from Directorate General of Family Planning facilities raises concerns about the accuracy and interpretation of data, particularly regarding the distinction between service unavailability and lack of service recipients. This issue has direct implications for women's health as it can mislead national-level managers and policymakers in evaluating the performance of Directorate General of Family Planning services and making informed decisions regarding family planning programmes, which are essential components of reproductive health initiatives aimed at improving women's health outcomes.

The study's identification of outliers within the WHO standard level emphasises the importance of further investigation and verification during data quality checks and supervision visits by facility managers and supervisors. Ensuring accurate and reliable data are crucial for assessing the effectiveness of reproductive health programmes and interventions targeting women's health.

Additionally, maintaining consistency between indicators and high accuracy rates across all tiers of health facilities, as highlighted in the study, is promising for data reliability and reflects positively on the overall quality of reproductive, maternal, new-born, and child health data. This reliability is essential for informed decision-making and policy formulation aimed at addressing reproductive health challenges and ultimately improving women's health outcomes. Therefore, addressing issues related to data accuracy and interpretation within the context of reproductive health is critical for advancing women's health initiatives and achieving broader health goals.

The findings of this study reveal important insights into reproductive health issues within the context of data reporting and accuracy. While inter-indicator consistency was generally maintained among most data from facilities under both Directorate General of Health Services and Directorate General of Family Planning, the least accurately reported indicator, postpartum family planning (PPFP) method use, highlights a specific area requiring improvement in data reporting and accuracy within the realm of reproductive health. Similarly, the absence of reported maternal deaths from sampled upazila health complexes despite expectations based on Bangladesh's health system structure raises questions about the effectiveness of the reporting system in capturing critical reproductive health data.

Moreover, Informal interviews revealing discrepancies in reporting structures and lack of specific training on data reporting systems among health facility personnel underscore the importance of capacity-building initiatives and standardised reporting protocols in ensuring consistent and accurate reproductive, maternal, new-born, and child health (RMNCH) data collection and reporting. These findings shed light on the challenges within the reproductive health data landscape and emphasise the significance of addressing issues related to data quality and reporting protocols to effectively monitor and improve reproductive health outcomes in Bangladesh, ultimately contributing to broader goals related to women’s health.

### Strengths

In this study, facilities from all tiers across all eight divisions, covering diverse geographical locations of the country, are evaluated. In addition to collecting data from documents at each facility, informal discussions were conducted to explore the structure of the existing data reporting system. This step was deemed necessary to gain a comprehensive understanding of the underlying dynamics and operation of the system.

### Limitations

WHO guidelines highlight two primary dimensions of data quality assurance: completeness and timeliness. Timeliness is generally assessed based on whether reports are submitted by facilities within set deadlines [32]. However, since this study did not involve real-time, monthly data extraction, we were unable to evaluate timeliness.

The management information system server of the Directorate General of Family Planning automatically records a zero (0) for any blank entry, regardless of whether the service was actually unavailable at the facility. Consequently, it was not possible to differentiate between true service unavailability and the lack of service receipt, making it difficult to identify actual missing values in the management information system server data.

To gain insights into the reporting process and associated challenges, informal discussions were conducted with reporting personnel. Although a thorough qualitative interview would have provided more comprehensive insights, time constraints of the focal person led to the exclusion of in-depth interviews from the study methodology.

### Recommendations

To enhance data reporting in Directorate General of Health Services and Directorate General of Family Planning, it is essential to establish a unified reporting system and appoint dedicated reporting focal persons at all levels of health facilities under both the Directorate General of Health Services and Directorate General of Family Planning. Additionally, comprehensive guidelines with operational definitions for all indicators must be developed and widely distributed to service providers and reporting focal persons across all Directorate General of Health Services and Directorate General of Family Planning facilities to ensure standardised reporting practices.

Furthermore, providing thorough training on digital data reporting systems and indicator definitions to all reporting focal persons within Directorate General of Health Services and Directorate General of Family Planning health facilities is necessary to enhance their capacity and accuracy in reporting. Ensuring the availability of functional laptops and internet connections at all levels of Directorate General of Health Services and Directorate General of Family Planning health facilities is also vital to facilitate timely and efficient reporting.

Moreover, transitioning to real-time data input instead of defaulting to reporting zero (0) in the Directorate General of Family Planning database would significantly improve the accuracy and timeliness of data reporting. Specialised sensitisation and training programmes should be implemented for reporting focal persons at UHCs to address their low completeness rate in indicator reporting.

Finally, conducting sensitisation programmes for reporting focal persons across most Directorate General of Health Services and Directorate General of Family Planning facilities is essential. These programmes should focus on accurately reporting two critical indicators: forceps/vacuum/breech delivery and septic abortion cases, ensuring the reliability and comprehensiveness of the reported data. It’s also crucial to foster a positive data use culture within Directorate General of Health Services and Directorate General of Family Planning through regular data quality assessments, dialogue sessions, and information sharing to support data-driven decision-making and policy formulation.

## CONCLUSIONS

This study emphasises the pivotal role of robust data reporting systems within health facilities, vital for generating accurate, timely information crucial for informed decision-making in public health. Rectifying deficiencies in reporting practices and offering comprehensive training to reporting personnel are fundamental measures to enhance data quality and integrity. Strengthening these aspects not only facilitates evidence-based decision-making but also fosters improved health outcomes and advances the broader goals of universal health coverage and sustainable development. Investing in the enhancement of data reporting systems is therefore imperative for optimising health care delivery and achieving meaningful progress in population health.

## Additional material


Online Supplementary Document
