# Supplementary material for: Assessing the quality of data for selected reproductive health indicators in designated public health facilities in Bangladesh
Source: J Glob Health. 2024 Dec 6;14:04259. doi: 10.7189/jogh.14.04259 (PMC11621577; doi:10.7189/jogh.14.04259)
Supplement: Online Supplementary Document [file jogh-14-04259-s001.pdf]

Additional Material
Online supplementary Document

Supplementary Table S.1: List of the DGHS health facilities

Division	District	District level	Upazila level	Community level
		District Hospital/ Medical College Hospital	UHC	Community Clinic
Dhaka	Manikganj	Manikganj 250 Bedded District Hospital	Ghior UHC	Pukhuria CC - Ghior
	Narayanganj	Narayanganj General (Victoria) Hospital	Araihazar UHC	Panchbariya CC - Araihazar
Barishal	Bhola	Bhola 250 Bedded District Sadar Hospital	Daulatkhan UHC	Charkhalifa Kalu Mridha Bari shanglagna CC**
	Patuakhali	Patuakhali 250 Bedded Sadar Hospital	Mirzaganj UHC**	Kakrabunia CC, Mirzaganj**
Khulna	Narail	Narail District Hospital	Lohagara UHC	Lutiya CC, Lohagara
	Jhenaidaha	Jhenaidah 250 Bedded General Hospital	Kaliganj UHC	Bharasimla CC, Kaliganj
Rajshahi	Joypurhat	Joypurhat 250 Bedded District Hospital	Khetlal UHC	Alampur CC, Kheatlal
	Chapai-nawabganj	Chapainawabganj 250 Bedded District Hospital	Shibganj UHC	Candipur CC, Shibganj*
Mymensingh	Sherpur	Sherpur 250 Bedded District Hospital**	Nakla UHC	Danakusha CC, Nakla
	Mymensingh	Mymensingh Medical College Hospital	Bhaluka UHC	Jamirdiya CC, Habirbari*
Rangpur	Lalmonirhat	Lalmonirhat District Hospital	Paatgram UHC	Bangkanda CC, Paatgram*
	Rangpur	Rangpur Medical College Hospital	Mithapukur UHC	Rameshbarpara CC, Mithapukur*
Chattogram	Brahmanbaria	Brahmanbaria 250 Bedded District Sadar Hospital	Sarail UHC	Shahbazpur Uttor CC - Sarail
	Khagrachari	Khagrachari District Hospital	Ramgarh UHC	Kagrabil CC, Ramgrah
Sylhet	Habiganj	Habiganj 250 Bedded District Hospital	Ajmiriganj UHC	Abdullahpur CC*
	Sunamganj	Sunamganj 250 Bedded District Sadar Hospital	Tahirpur UHC	Daksinkul CC-Tahirpur

**Not functioning and permanently closed (found during field visit)*
***Do not practice paper-based reporting system*

Supplementary Table S.2: List of the DGFP health facilities

Division	District level		Upazila level		Union level
	District	MCWC	Sadar Clinic	MCH-FP	UH&FWC
Dhaka	Manikganj	Manikganj Sadar MCWC	Ghior Sadar Clinic	MCH-FP Unit, Ghior †	Baliyakhora UH&FWC
	Narayanganj	Narayangonj Sadar MCWC	Araihazar Sadar Clinic	MCH-FP Unit, Araihazar	Duptara UH&FWC
Barisal	Bhola	Bhola Sadar MCWC	Daulatkhan Sadar Clinic	MCH-FP Unit, Daulatkhan*	Charkhalifa UH&FWC
	Patuakhali	Patuakhali Sadar MCWC	Mirzaganj Sadar Clinic	MCH-FP Unit, Mirzaganj	Karabunia UH&FWC
Khulna	Narail	Narail Sadar MCWC	Lohagara Sadar Clinic	MCH-FP Unit, Lohagara†	Dighalia UH&FWC
	Jhenaidaha	Jheniadha MCWC	Kaliganj Sadar Clinic	MCH-FP Unit, Kaliganj†	Simla-Rokonpur UH&FWC
Rajshahi	Joypurhat	Joypurhat Sadar MCWC	Khetlal Sadar Clinic	MCH-FP Unit, Khetlal	Alampur UH&FWC
	Chapai-nawabganj	Nawabgonj Sadar MCWC	Shibganj Sadar Clinic	MCH-FP Unit, Shibganj	Jhilim UH&FWC
Mymensingh	Sherpur	Sherpur Sadar MCWC	Nakla Sadar Clinic	MCH-FP Unit, Nakla†	Nakla UH&FWC
	Mymensingh	Mymensingh Sadar MCWC	Bhaluka Sadar Clinic	MCH-FP Unit, Bhaluka	Habir bari UH&FWC
Rangpur	Lalmonirhat	Lalmonirhat Sadar MCWC	Patgram Sadar Clinic	MCH-FP Unit, Patgram†	Patgram UH&FWC*
	Rangpur	Rangpur Sadar MCWC	Durgapur (Mithapukur) Sadar Clinic	MCH-FP Unit, Mithapukur†	Bara Hajratpur UH&FWC
Chattogram	Brahmanbaria	Brahmanbaria Sadar MCWC	Sarail Sadar Clinic	MCH-FP Unit, Sarail	Aruail UH&FWC
	Khagrachari	Khagrachari Sadar MCWC	Ramgarh Sadar Clinic	MCH-FP Unit, Ramgarh	Patachhara UH&FWC
Sylhet	Habiganj	Habiganj Sadar MCWC	Ajmiriganj Sadar Clinic	MCH-FP Unit, Ajmiriganj†	Shibpasha UH&FWC
	Sunamganj	Durgapasa Sadar MCWC	Tahirpur Sadar Clinic	MCH-FP Unit, Tahirpur	Uttar Sreepur UH&FWC

**Not functioning and permanently closed (found during field visit)*
†conjugated with the Sadar Clinics of the same sub-district & reporting as a single facility
